# Detection of bluetongue virus in *Culicoides* spp. in southern Yunnan Province, China

**DOI:** 10.1186/s13071-020-04518-z

**Published:** 2021-01-22

**Authors:** Ying Liang Duan, Le Li, Glenn Bellis, Zhen Xing Yang, Hua Chun Li

**Affiliations:** 1 Yunnan Tropical and Subtropical Animal Virus Diseases Laboratory, Yunnan Animal Science and Veterinary Institute, Kunming, Yunnan Province China; 2 Research Institute for the Environment and Livelihoods, Charles Darwin University, Darwin, NT Australia; 3 Department of Agriculture, Water and the Environment, Darwin, NT Australia

**Keywords:** *Culicoides*, Bluetongue virus vector, *C. jacobsoni*, *C. tainanus*, *C. imicola*

## Abstract

**Background:**

*Culicoides* (Diptera: Ceratopogonidae) are vectors for many arboviruses. At least 20 species are considered as vectors or potential vectors of bluetongue virus (BTV) which cause bluetongue disease in ruminants. A BTV prevalence of 30–50% among cattle and goats in tropical southern Yunnan Province, China, prompted an investigation of the potential BTV vectors in this area.

**Methods:**

*Culicoides* were collected by light trapping at three sites in the tropical region of Yunnan Province. Species were identified based on morphology and DNA sequences of cytochrome* c* oxidase subunit 1 (*cox*1). PCR and quantitative PCR following reverse transcription were used to test for the presence of BTV RNA in these specimens. Phylogenetic analysis was used to analyze the *cox*1 sequences of *Culicoides* specimens infected with BTV.

**Results:**

Approximately 67,000 specimens of *Culicoides* were collected, of which 748 were tested for the presence of BTV. Five specimens, including two of *Culicoides jacobsoni*, one of *C. tainanus* and two of *C. imicola*, were identified as infected with BTV. No specimens of *C*. (subgenus *Trithecoides*) or *C. oxystoma* tested were positive for BTV infection.

**Conclusions:**

To our knowledge this is the first report of *C. jacobsoni* as a potential BTV vector and the fourth report of an association between *C. tainanus* and BTV, as well as the first direct evidence of an association between BTV and *C. imicola* in Asia. A fourth potential cryptic species within *C. tainanus* was identified in this study. Further analysis is required to confirm the importance of *C. jacobsoni* and *C. tainanus* in BTV epidemiology in Asia. 
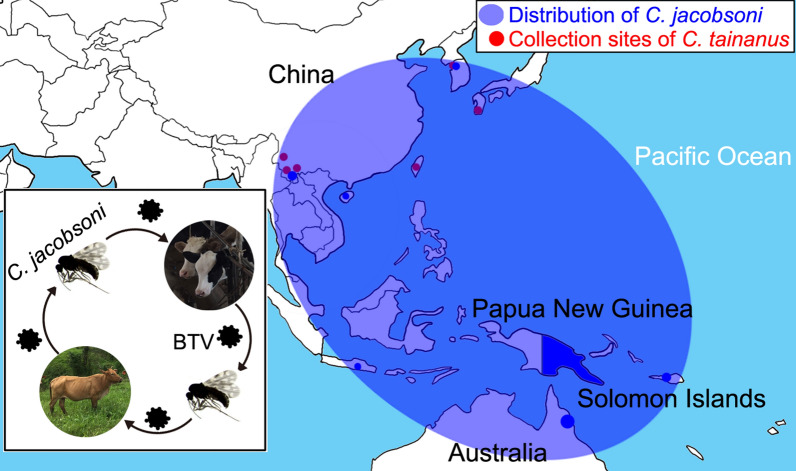

## Background

Members of genus* Culicoides* (Diptera: Ceratopogonidae) are vectors for many arboviruses, and at least 50 viruses belonging to families *Bunyaviridae*, *Reoviridae*, and *Rhabdoviridae* have been associated with several species of *Culicoides* in numerous countries [1, 2]. The genus *Culicoides* contains 1347 species placed into 33 subgenera and 38 species groups [3, 4]. More than 40 species are considered to be vectors or potential vectors of pathogens, with at least 20 of these being associated with bluetongue virus (BTV) [2, 5, 6]. However, the species serving as vectors of BTV differ on different continents, indicating that it is meaningful to investigate local BTV vectors.

 Bluetongue disease, a serious communicable disease among ruminants such as sheep, goats, and cattle, is caused by BTV (genus *Orbivirus*, family *Reoviridae*). Bluetongue disease was first recognized in 1876 in South Africa [7]. The first detection of BTV in China was reported from Shizong, Yunnan Province, in 1979 [8], and 14 serotypes (BTV 1, 2, 3, 4, 5, 7, 9, 11, 12, 15, 16, 21, 23, and 24) have since been identified in China [9, 10]. A BTV prevalence of 30–50% among cattle and goats has been reported in the tropical southern part of Yunnan Province [11, 12], indicating an active cycle of infection and the presence of competent vectors.

Very little attention has been focused on investigating the vectors of BTV in Asia. Several species that are known to act as vectors in other countries, such as *Culicoides imicola* Kieffer,* C. brevitarsis* Kieffer,* C. fulvus* Sen & Das Gupta, *C. actoni* Smith,* C. wadai* Kitaoka,* C. obsoletus* (Meigen) and *C. pulicaris* (L.), have been reported from Asia [13–15], but direct evidence from Asian populations is lacking for most of these species. Extrapolation of results from other countries can, however, be problematic as the taxonomy of several of these species has not been fully resolved and several have been shown to be complexes of cryptic species that may not have similar vector capacity [16, 17]. Recent studies have detected BTV in field-collected specimens of at least four *Culicoides* species in Asia, namely *C. asiana* Bellis, *C.* sp. near *obsoletus*,* C. tainanus* Kieffer and *C*. sp near *tainanus* [18–20], suggesting these species may be involved in BTV transmission.

Although BTV is endemic in Yunnan, there is currently little information available on the species that are acting as vectors. Several of the species mentioned in the preceding text are known to occur in Yunnan [13] but there has been only investigation into their role as vectors conducted in the high-altitude county of Shangrila [19] where BTV prevalence is relatively low [10]. Tropical parts of Yunnan Province share a border with Myanmar, Lao and Vietnam, and knowledge of the vector species in the tropical areas of Yunnan could therefore be useful in improving our understanding of the epidemiology of BTV and other arborviruses, such as the recently arrived African Horse Sickness virus, in southern Asia. Here, we report on the detection of BTV virus in specimens of *Culicoides* collected from tropical areas of Yunnan Province.

## Methods

### *Culicoides* collection method

**Fig. 1:**
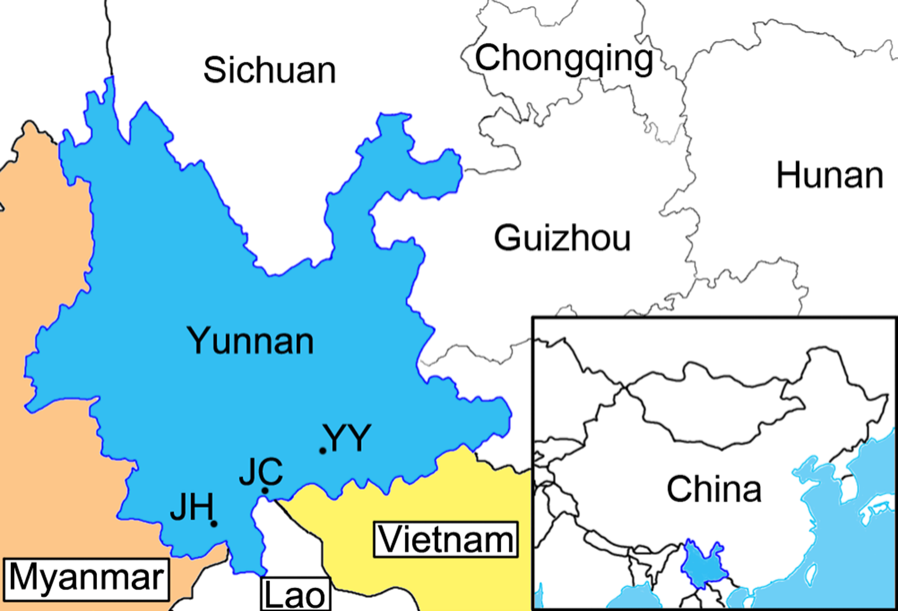
Local map of the three collection sites in southern Yunnan Province, China.* JC* Jiangcheng, *JH* Jinghong, *YY* Yuanyang

*Culicoides* were collected from livestock farms near three cities in southern Yunnan Province, China (Table 1). These cities, namely Jinghong, Jiangcheng, and Yuanyang, are located close to the Myanmar, Lao and Vietnam borders, respectively (Fig. 1).

**Table 1:** Location and type of farm where *Culicoides* were collected

City	Date	Site	Exact coordinates		
			Latitude (°N)	Longitude (°E)	Elevation (m a.s.l.)
Jinghong	(i) 8–9 Jul 2019 (ii)14–15 Nov 2019	15 buffaloes	21.52	100.64	726
Jiangcheng	4–5 Jul 2019	50 cattle	22.60	101.85	1091
Yuanyang	5–6 Aug 2018	100 cattle; 80 goats	23.24 23.24	102.79 102.81	183 183

Battery-powered UV light traps (LTS-M02; Wuhan Lucky Star Medical Treatment Technology Co., Wuhan, China) were set inside livestock farms, 5–10 meters from penned cattle, buffalo or goats. Two traps were set at each farm for a single night (5 pm to 9 am next day) (Table 1). Specimens were collected dry into mesh bags and transferred to 90% ethanol immediately after clearing the trap.

### Morphological identification

Specimens were identified by morphology under a dissecting microscope according to the keys and descriptions of Liu et al. [13], Wirth and Hubert [15], and Bellis [21]. Many specimens belonging to *C.* subgenus *Trithecoides* could not be reliably identified to species unless mounted onto slides, which was impractical for most specimens; consequently the specimens were sorted into two pools of specimens, those with a uniformly yellow scutum and those with a yellow scutum with brown anterior markings. Representative specimens of each species and all specimens which were identified as being infected with BTV were mounted onto microscope slides following non-destructive nucleic acid extraction using the methods of Bellis et al. [22].

### Nucleic acid extraction

Because the insects were collected dry, the abdomen of many specimens were partially shrivelled and it was difficult to be certain of parity [23]; therefore, no attempt was made to separate nulliparous from parous specimens. However, most specimens with traces of a blood meal were removed from further processing. Mature female specimens were submitted for individual non-destructive nucleic acid extraction using a procedure modified from the method of Duan et al. [19]. Briefly, each midge was incubated in 50 μl lysis buffer from the Genomic DNA Extraction kit (DP304; TIANGEN, Tiangen, Beijing, China) at 30 °C for 16 h. Subsequently, 30 μl of prepared lysis sample was used to extract nucleic acids. The DNA and RNA were extracted together using a MagMAX™M-96 Viral RNA Isolation kit (Ambion®, Thermo Fisher Scientific, Waltham, MA, USA) following the manufacturer’s directions and a MagMAX™ Express-96 machine (Ambion®, Thermo Fisher Scientific). The nucleic acids were eluted with 50 μl of elution buffer and stored at − 20 °C until use.

### Reverse transcription-quantitative PCR and reverse transcriptase-PCR

It was anticipated that most midges would not be infected with virus so a method of screening large numbers of specimens was devised that still allowed subsequent analysis of individual specimens. A 10-μl aliquot of lysate was taken from eight individual specimens and pooled into an 80-μl sample which was submitted for reverse transcription-quantitative PCR (RT-qPCR). Any pool with a quantification cycle (Cq) value of < 35 might contain positive samples; therefore, a 30-μl aliquot of lysate from each of the eight specimens from positive pools (Cq < 35) was processed individually using the same RT-qPCR. Lysate from individual specimens which produced a Cq value of < 25 were regarded as being infected.

A RT-qPCR using the primers BTVF-MH and BTVR-MH and probe BTVP-MH targeting the BTV seg10, as described by Hofmann et al. [24], was used to detect BTV RNA. Briefly, the reaction solution was prepared using the Quant One Step RT-qPCR kit (TIANGEN) according to the manufacturer’s instructions, and 20 µl was added to 2 µl of RNA sample. The RT-qPCR was performed on a Fast7500 Realtime PCR machine (Applied Biosystems, Foster City, CA, USA) at the following cycling conditions: 45 °C, 10 min; 95 °C, 2 min; 95 °C/10 s for 45 cycles; and a final extension at 65 °C for 45 s. Fluorescence was measured at the end of each extension step.

Serotype-specific RT-qPCR tests employing primers developed from local viruses and targeting BTV seg2 of 12 serotypes (BTV-1, BTV-2, BTV-3, BTV-4, BTV-5, BTV-7, BTV-9, BTV-12, BTV-15, BTV-16, BTV-21, BTV-24) (Song et al. in review) were utilized to confirm the primary detection of BTV RNA. The RT-qPCR was run in two steps. In the first step, viral cDNAs were synthesized using the Reverse Transcriptase M-MLV kit (Takara, Osaka, Japan) with random 6-mer primers. Each reaction mixture contained 12 μl of specimen RNA and 12 μl of reverse transcription solution. The cycling conditions were: 30 °C, 5 min; 45 °C, 35 min; then 72 °C, 10 min. In the second step, 2 μl of cDNA template was detected by qPCR in 20 μl of solution, using the PrimeDirect™ Probe RT-qPCR kit (Takara) following the manufacturer’s instructions. The qPCR program consisted of: 95 °C, 30 s; then 95 °C/5 s, 60 °C/34 s for 40 cycles. Fluorescence signals were recorded after each extension step.

A second RT-PCR test employing primers BTV-VP1-pan-F (5′-ATGCAATGGT CGCAATCACC G) and BTV-VP1-B0-R (5′-TTTCGAATAT GGGCAGCCT) and targeted BTV seg1 of BTV-16, BTV-20, and BTV-23 was used to confirm the presence of BTV in the positive specimen of *C. tainanus* JCP4-5E. A 5-μl aliquot of cDNA from this specimen was added to a PrimeSTAR® GXL (Takara) PCR system with a final volume of 50 μl. The PCR cycling conditions were: 95 °C, 1 min; then 95 °C/10s, 55 °C/10 s, 68 °C/1 min for 40 cycles. The amplified segments were sequenced using primers BTV-VP1-pan-F and BTV-VP1-B0-R.

### Amplification and sequencing of *Culicoides *cytochrome* c* oxidase subunit 1 gene

The identification of specimens identified as being infected with BTV was confirmed by amplification of the cytochrome* c* oxidase subunit 1 (*cox*1) gene using a method modified from Duan et al. [19]. Briefly, 5.5 μl of DNA was added to 14.5 μl of reaction solution prepared using the PrimeSTAR® GXL kit (Takara) and primers BC1culicFm and JerR2m of Bellis et al. [25]. PCR cycling conditions were: 95 °C, 2 min; then 95 °C/10 s, 45 °C/10 s, 68 °C/50 s for 30 cycles; with a final extension at 68 °C for 30 s, followed by incubation at 4 °C. Subsequently, 10 μl of fresh 1× PCR solution was added to each tube and a second round of PCR was run as described above for 22 cycles. PCR products were sent to Kunming Shuoqing Biological Technology Company (China) for Sanger sequencing with an ABI3739XL machine (Applied Biosystems). The 646-bp fragments just between the 3′ ends of the primers were assembled and used for sequence analysis.

### *Cox*1 sequence analysis

*Cox*1 sequences from specimens infected with BTV and those of two conspecific specimens of *C. tainanus* were uploaded to the National Center for Biotechnology Information (NCBI), and the top 250 closest matched sequences were located using the Basic Local Alignment Search Tool (BLAST). Sequences with coverage of < 90%, lacking country information or not having a firm identification were omitted, as were any redundant records from the same submission batch. Selected homologous sequences were downloaded and the extrusive 5′ end and 3′ end of the sequences were truncated and aligned with our sequences using the MUSCLE sequence alignment of MEGA-X with default parameters. Phylogenetic trees were constructed using the neighbor-joining method (model = Kimura 2, bootstrap = 1000). Sequence alignment and phylogenetic tree building were completed uising MEGA-X software.

## Results

### Screening *Culicoides* by RT-qPCR

Approximately 67,000 specimens were collected from the three sites (Fig. 1; Table 2). The diversity and abundance of these species will be reported in a future publication. A total of 748 specimens comprising 11 morphologically recognizable species were pooled into 94 pools and tested for the presence of BTV (Table 3). The actual number of species is, however, greater than this as several species have since been shown by *cox*1 analysis to comprise more than one cryptic species (data not shown).

**Table 2:** Relative abundance of *Culicoides* species at the three collection sites in southern Yunnan Province, China

Location (collection time or animal)	Dominant species			No. of collected *Culicoides *^a^
	Most dominant	Second most dominant	Third most dominant	
Jinghong (Jul)	*C. oxystoma* (95.2%)	*Trithecoides* (2.4%)	*C. innoxius* (1.4%)	842/5000
Jinghong (Nov)	*C. oxystoma* (52.4%)	*Trithecoides* (20.1%)	*C. jacobsoni* (13.9%)	288/300
Jiangcheng	*Trithecoides* (92.5%)	*C. spiculae* (4.1%)	*C. tainanus* (2.0%)	1222/55,000
Yuanyang (cattle)	*C. oxystoma* (42.5%)	*C. jacobsoni* (27.7%)	*C. arakawai* (7.6%)	232/3600
Yuanyang (goats)	*C. imicola* (42.1%)	*C. innoxius* (20.7%)	*C. arakawai* (14.7%)	287/3, 400
Total	*Trithecoides* (76.8%)	*C. oxystoma* (10.1%)	*C. spiculae* (3.3%)	2871/67,000

*Trithecoides* in table refers to species belonging to *C. *subgenus *Trithecoides* complex with yellow scutum, which includes *C. flavescens*, *C. fordae*, *C. palpifer* and one other, probably undescribed species

^a^Numbers shown are the number of specimens sorted compared to the total estimated to be present in each collection

**Table 3:** Detection of bluetongue virus seg10 RNA by reverse transcription-quantitative PCR from individual specimens of *Culicoides* species from Yunnan Province, China

Species	Source^a^	Number of midges tested	RT-qPCR test results (values are number of midges)^b^				Percentage of infected specimens (Cq ≤ 25)
		Total	Cq ≤ 25	25 < Cq ≤ 30	Cq > 30	Non- reactive	
*C. jacobsoni*	JH, YY	85	2	0	0	83	2.35
*C. imicola*	YY	40	2	0	0	38	5.0
*C. tainanus*	JC	105	1	0	3	101	0.95
*Trithecoides*	JH, JC, YY	250	0	0	1	249	0
*C. oxystoma*	JH, JC, YY	105	0	0	0	105	0
*C. arakawai*	JH, JC, YY	60	0	0	0	60	0
*C. orientalis*	JH, JC, YY	38	0	0	2	36	0
*C. guttifer*	JH, JC	31	0	0	0	31	0
*C. spiculae*	JC	24	0	0	2	22	0
*C. insignipennis*	JH	8	0	0	0	8	0
*C. liui*	JH	2	0	0	0	2	0
Total		748	5	0	8	748	0.67

RT-qPCR, Reverse transcription-quantitative PCR

^a^JC, Jiangcheng; JH, Jinghong; YY, Yuanyang

^b^From a pool of 8 specimens with a collective quantification cycle (Cq) value of < 35, indicating possible positivity, individual specimens were tested, using the same RT-qPCR. Lysate from individual specimens which produced a Cq value of < 25 was considered to indicate positivity for infection. Numbers indicate the number of specimens testing at the indicated Cq

Of the 94 pools tested, only 13 were found to have a Cq value < 35, and only five of the individual specimens in those pools exhibited Cq values sufficiently low enough (Cq < 25) to suspect the insects were infected with BTV. Of the remaining individual specimens from the pools, eight exhibited Cq > 30, and the remainder did not react (these these were all considered to be non-infected midges).

The five BTV-positive specimens included two specimens morphologically identified as *C. jacobsoni*, two identified as *C. imicola*, and one identified as *C. tainanus*; these specimens came from Jinghong, Yuanyang, and Jiangcheng, respectively (Table 4). *Culicoides jacobsoni* comprised approximately 13.9% and 14.8% of the total catch from Jinghong (November) and Yuanyang, respectively; *C. tainanus* comprised approximately 0.3% and 2% of the total from Jinghong (November) and Jiangcheng, respectively; and *C. imicola* comprised approximately 0.6% and 21.6% of the total from Jinghong (July) and Yuanyang, respectively.

**Table 4:** Genbank accession numbers and genetic similarity of the *Culicoides* specimens from Yunnan Province, China, found to be infected with bluetongue virus

Specimen voucher	Species	Place of origin	GenBank accession No.	Nearest record on GenBank		BTV detection	
				Accession No.	Similarity (%)	Cq	Serotype
P1311	*C. jacobsoni*	Jinghong	MT576581	KF297817.1	99.54	24.2	4
P1318	*C. jacobsoni*	Jinghong	MT576582	KT352352.1	99.23	23.8	4
JCP4-5E	*C. tainanus*	Jiangcheng	MT576577	AB360987.1	90.77	22.7	Uncertain
P1B44	*C. imicola*	Yuanyang	MT576579	KT307820.1	100	21.0	15
P1D52	*C. imicola*	Yuanyang	MT576580	KT307820.1	99.85	24.5	16

BTV, Bluetongue virus

### BTV serotype confirmation

The two specimens of *C. jacobsoni* and two of *C. imicola* that were identified as being infected with BTV in the group test exhibited Cq of between 30 and 34 in the serotype-specific test reacting to serotype 4, 4, 15, and 16, respectively (Table 4). The sample from *C. tainanus* (JCP4-5E) was not amplified by any of the serotype-specific RT-qPCR reactions. However, a BTV seg1 fragment was amplified successfully from this sample, and the 923-bp sequence (NCBI: MT757691) showed 97.19% homogeneity with a sequence from India BTV-1 (KP696562.1 and KP696552.1) and 96.54% homogeneity with a sequence of BTV-16 (MH990415.1 and KP820871.1).

### Identification of the BTV-infected *Culicoides*

*Cox1* sequences of 646 bp from the five BTV-infected specimens and from two *C. tainanus* were used for BLAST analysis on NCBI (Table 4). The closest matches to our two *C. jacobsoni* (P1311 and P1318) were specimens of *C. jacobsoni* from South Korea (KF297817.1) and the Solomon Islands (KT352352.1); the nearest match with our *C. tainanus* (JCP4-5E) is a specimen of *C. maculatus* Shiraki (now synonymized with *C. tainanus*) from Japan (AB360987.1); and the closest match to our *C. imicola* (P1B44 and P1D52) is a specimen of *C. imicola* from India (KT307820.1).

### Phylogenetic analysis of the BTV-infected *Culicoides*

*Cox1* sequences from these specimens were truncated to provide 100% coverage with homologous sequences downloaded from NCBI. This resulted in 623-bp fragments of *C. jacobsoni*, 555-bp fragments of *C. imicola*, and 433-bp fragments of *C. tainanus*. These fragments were used to construct phylogenetic trees with the sequences downloaded from NCBI.

**Fig. 2:**
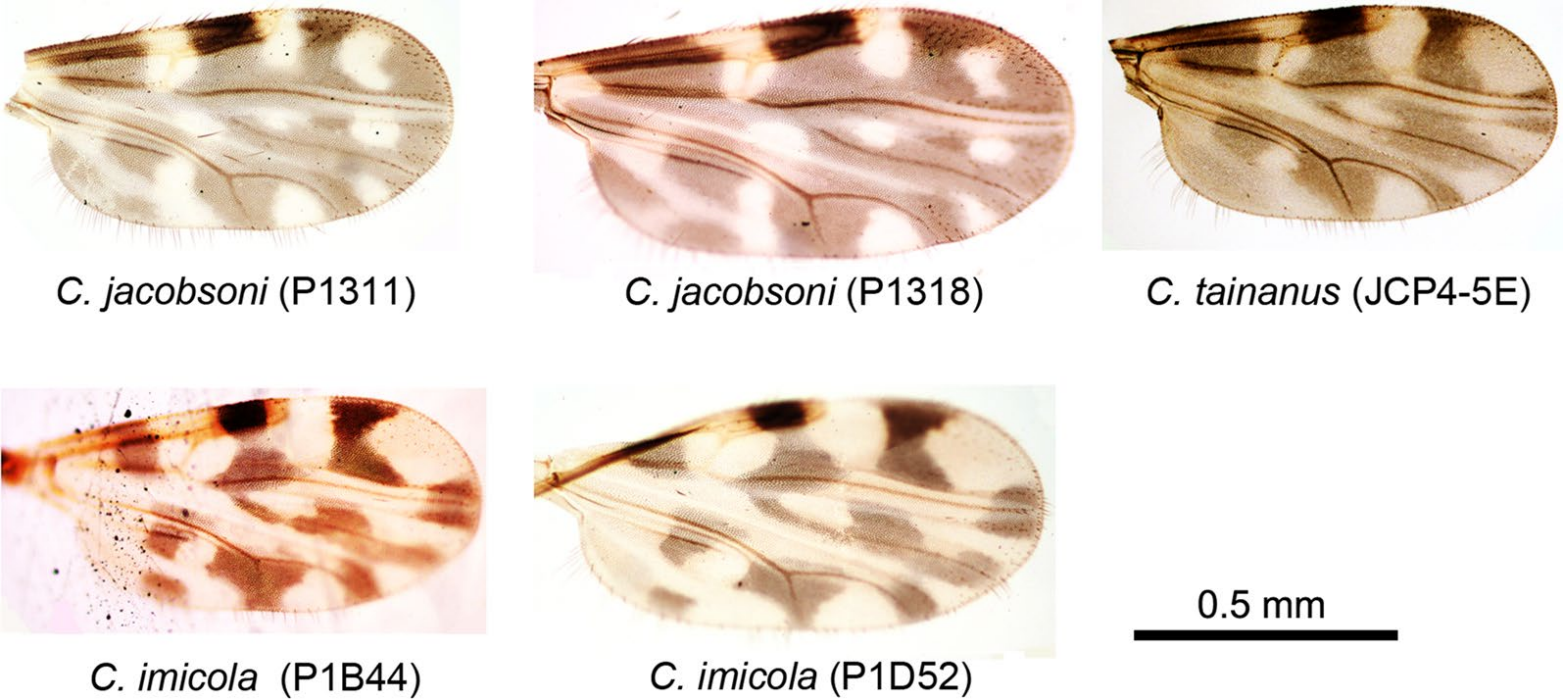
Wings of the five specimens of *Culicoides* found to be infected with bluetongue virus (BTV). Scale bar: 0.5 mm

**Fig. 3:**
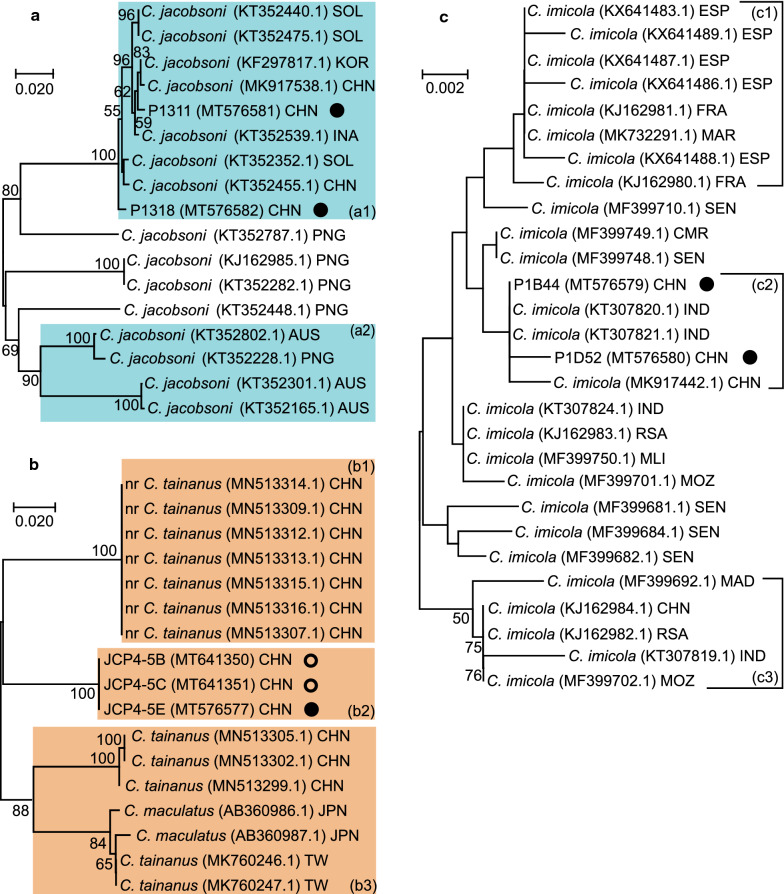
Phylogenetic analysis of three *Culicoides* species found to be infected with BTV. Neighbor-joining (NJ) trees of pairwise genetic distances among cytochrome* c* coxidase subunit 1 (*cox1*) sequences from the BTV-infected specimens (labeled by solid black circles) and their closest matches from NCBI. Three NJ trees were built for *C. jacobsoni* (**a**), *C. tainanus* complex (**b**), and *C. imicola* (**c**), respectively. Bootstrap values < 50% were omitted. For each specimen, species name, Genbank accession number, and the country/region of collection are provided. Some clades are highlighted in different colours. The specimens created in this study are labeled by circles. Solid black circles indicate specimens shown to be infected with BTV. Hollow black circles indicate non-infected sepciemns of *C. tainanus *used for comparative purposes. *AUS* Australia, *CHN* China, *CMR* Cameroon, *ESP* Spain, *FRA* France, *INA* Indonesia, *IND* India, *JPN* Japan, *KOR* South Korea, *MAD* Madagascar, *MAR* Morocco, *MLI* Mali, *MOZ* Mozambique, *MRI* Mauritius, *PNG* Papua New Guinea, *RSA* South Africa, *SEN* Senegal, *SOL* Solomon Islands, *TLS* Timor-Leste, *TW*Taiwan.

As shown in Fig. 3a, Papua New Guinea (PNG) has an abundant variety of *C. jacobsoni*. Gopurenko et al. [17] reported large genetic variation within *C. jacobsoni*, and our specimens fall into one of the clades they reported. Specimens belonging to this clade were shown to be present in China, the Solomon Islands (SOL), Indonesia (INA), and South Korea (KOR). Another specimen belongs to a clade from Australia (a2) (Fig. 2).

Phylogenetic analysis of specimens identified as *C. tainanus* indicated the presence of four clades with a minimum distance of 9.70% separation (Fig. 3b).

The phylogenetic tree of *C. imicola* is shown in Fig. 3c. Although several clades appear evident, their genetic distance is small, with a maximum distance of 1.24%.

## Discussion

To the best our our knowledge, this is the first report of an association between *C. jacobsoni* and BTV. This *Culicoides* species, which belongs to *C.* subgenus *Avaritia*, a subgenus that contains a high proportion of vector species [2], has recently been associated with other bovine arboviruses, such as Peaton virus [18] and Akabane virus [26], among others. The detection of BTV in *C. imicola* is not surprising as this species is a proven vector in Africa and the Mediterranean [5] and is one of the dominant *Culicoides* species in southern India [27]. The detection of BTV in specimens of this species from China appears to be the first evidence of a direct association between BTV and *C. imicola* in Asia. Similarly, the detection of BTV from *C. tainanus* represents the fourth report of an association of this species with BTV [18, 19] which, combined with reports on its dominance in collections from cattle farms [28–30], suggests this species may be an important vector of BTV in the region. The absence of infected specimens from 250 specimens of *C*. subgenus *Trithecoides* and 105 specimens of *C. oxystoma* (Table 3) would appear to suggest that these species are less important in terms of BTV epidemiology, but as the proportion of these specimens that were parous is unknown, it is difficult to draw conclusions about these negative results. Similarly, no conclusions may be drawn from the negative results for the other species tested as too few specimens were processed.

**Fig. 4:**
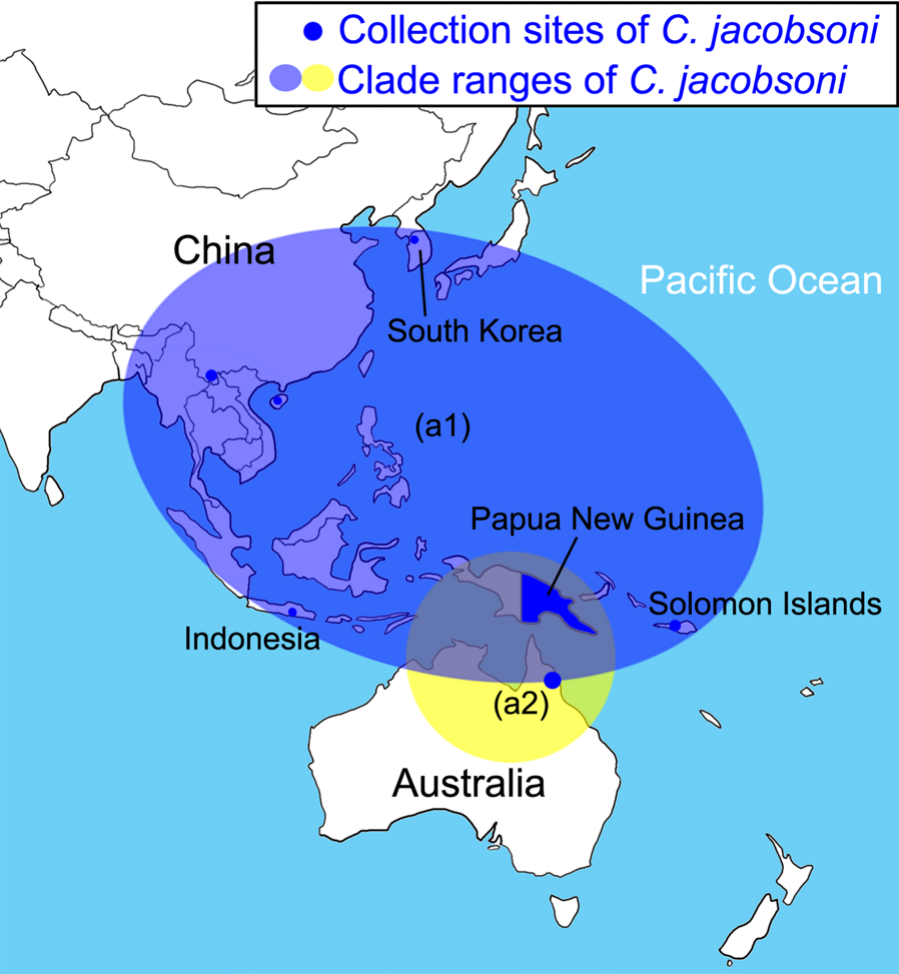
Partial distributions of *C. jacobsoni* in map draft. Filled blue circles indicate the collection sites of the *C. jacobsoni* specimens used in the phylogenetic analysis. Estimated distribution regions of the major clades are highlighted in yellow and blue shading, respectively. The size of the circles are proportional to the sample numbers. See Fig. 3 for structure of clade (a1, a2)

Gopurenko et al. [17] reported numerous cryptic species of *C. jacobsoni* in Australasia and southern and eastern Asia and cited the need for further integrative taxonomic research to clarify the status of these cryptic species. The two specimens found here to be infected with BTV appear to be conspecific with one of these cryptic species with a Barcode Index Number (BIN) of AAI9869 which Gopurenko et al. [17] suggested is likely to be *C. jacobsoni* (*s.s.*) as it is the only species from this complex present in mainland Asia. This species is widespread throughout Asia and Australasia, and our records from Yunnan Province represent a significant western extension of this distribution (Figs. 3a, 4). The association of this species with BTV therefore contributes to current understanding of the epidemiology of this virus across the region, not just in Yunnan.

The phylogenetic analysis of *C. tainanus* (Fig. 3b) reveals the existence of four potential cryptic species, including the two recently reported by Duan et al. [19] from Shangri-la in the north of Yunnan. The specimen associated with BTV in the present study appears to belong to a new potential cryptic species that is 9% different to the others reported on NCBI (Table 4). All four of these potential cryptic species have now been associated with BTV; BIN AAI9872 from Taiwan and Japan [18]; BIN ADR0709 and BIN ADQ7496 from Shangri-la, Yunnan Province [19]; and BIN AEA8529 from the present study. To date, three of the four clades of this species are only known from Yunnan, but they may be more widely distributed, in which case these cryptic species may be associated with BTV in other areas as well. The status of these four phylogenetic clades requires investigation using the integrative taxonomic procedures suggested by Gopurenko et al. [17] to confirm the status of these putative cryptic species.

The phylogenetic tree of *C. imicola* showed little genetic diversity within this species, as has been reported elsewhere [31–33]. The specimens from Yunnan were most similar to specimens from India, although the *cox*1 sequences of *C. imcola* showed little variation despite the large geographic separation of the specimens analyzed (Fig. 3c).

The successful detection of the BTV seg2 from the specimens of *C. jacobsoni* and *C. imicola* and of the BTV seg1 from *C. tainanus* supports the conclusion that these specimens were indeed infected with BTV. The failed amplification of seg2 from the *C. tainanus* specimen is likely due to an inappropriate primer set for the strain of virus present in this insect.

Infection of vectors results in the multiplication of virus by several orders of magnitude as virus infects various organs, particularly the midgut and salivary glands [34]. The significantly higher titer of virus in infected individuals compared to non-infected allows the identification of these individuals using a RT-qPCR assay [35–37]. The bimodal pattern of Cq values observed in our study (Table 3) is similar to that observed in field-collected specimens reported by Duan et al. [19]. Taken together, these results support the conclusions of Veronesi et al. [35] and Van der saag et al. [36, 37].

Studies on the vector competence of *Culicoides* in many parts of the world, including Asia, have lagged behind those of other countries, partially due to the difficulties and expense of collecting midge specimens for virus isolation attempts. The recent development of RT-qPCR methods to screen potential vectors provides an economical alternative to traditional virological studies by using specimens preserved in ethanol that allow for subsequent morphological confirmation of identifications. This latter approach is emerging as an important issue, as several vector species are being revealed by molecular studies as complexes of cryptic species that will require detailed integrative taxonomic studies to resolve [17, 33].

## Conclusions

Three species belonging to *C. * subgenus* Avaritia* were found to be associated with BTV in the tropical area of Yunnan Province, China by RT-qPCR tests targeting BTV segments 1, 2, and 10. In this study, *C. jacobsoni* is reported as a potential BTV vector for the first time. We report for the fourth time an association between *C. tainanus* and BTV and provide the first hard evidence of an association between BTV and *C. imicola* in Asia. We also report the presence of a further potential cryptic species of *C. tainanus.*. These data suggest that *C. jacobsoni* and *C. tainanus* might be important to the epidemiology of BTV in East Asia.

## Data Availability

Data supporting the conclusions of this article are provided within the article. Raw data are available from the corresponding author upon request.

## Abbreviations

BIN: Barcode index numbers
BLAST: Basic local alignment search tool
BTV: Bluetongue virus
*cox*1: Cytochrome* c* oxidase subunit 1
Cq: Quantification cycle
NCBI: National Center for Biotechnology Information
NJ: Neighbor-joining method
RT-qPCR: Reverse transcription-quantitative PCR
